# The Alleviative Effect of Naringin Against Cardiovascular Dysfunction and Remodeling in Hypertensive Rats by Suppressing the Angiotensin II Pathway

**DOI:** 10.1002/fsn3.70484

**Published:** 2025-06-19

**Authors:** Juthamas Khamseekaew, Metee Iampanichakul, Prapassorn Potue, Putcharawipa Maneesai, Panot Tangsucharit, Siwayu Rattanakanokchai, Poungrat Pakdeechote

**Affiliations:** ^1^ Department of Physiology, Faculty of Medicine Khon Kaen University Khon Kaen Thailand; ^2^ Department of Pharmacology, Faculty of Medicine Khon Kaen University Khon Kaen Thailand; ^3^ Faculty of Veterinary Medicine Khon Kaen University Khon Kaen Thailand

**Keywords:** cardiovascular dysfunction and remodeling, hypertension, naringin, renin‐angiotensin system oxidative stress

## Abstract

Naringin is an essential citrus flavonoid with numerous biological benefits. However, its influence on the heart and aorta during hypertension is poorly understood. This study aimed to determine whether naringin could alleviate left ventricular (LV)‐aortic dysfunction and remodeling in hypertensive rats generated using *N*_ω_‐Nitro‐L‐arginine methyl ester hydrochloride (L‐NAME). Rats were concurrently administered L‐NAME (40 mg/kg body weight (BW)/day), telmisartan (5 mg/kg BW/day), or naringin (20 or 40 mg/kg BW/day) for the 5‐week trial. Similar to telmisartan, naringin prevented elevated blood pressure in L‐NAME‐treated rats (*p* < 0.05). Hypertensive rats showed reductions in LV fraction shortening and ejection fraction, which did not occur in the naringin‐treated group. Aortic endothelial function was attenuated in the hypertensive group compared to the naringin‐treated group (*p* < 0.0001). L‐NAME‐treated rats showed alterations in cardiovascular morphology, including LV aortic hypertrophy and fibrosis (*p* < 0.05), whereas the naringin‐treated group did not exhibit these changes. Hypertensive rats had greater concentrations of renin‐angiotensin system (RAS) markers, oxidants/antioxidant parameters, nitrate/nitrite, and tumor necrosis factor‐α in circulation than the naringin‐treated group (*p* < 0.05). Naringin attenuated the upregulation of angiotensin II receptor type I, protein kinase C, NADPH oxidase 2, Raf‐1, and extracellular signal‐regulated kinases 1/2 in the LV tissues of L‐NAME rats (*p* < 0.002). In conclusion, naringin alleviated LV aortic dysfunction and remodeling by suppressing RAS parameters, oxidative stress, inflammation, and restoring the AT1R/PKC/NOX2/Raf‐1/ERK1/2 signaling pathway. These findings suggest that naringin is a promising alternative for the management of hypertension.

## Graphical abstract

Naringin alleviated LV‐aortic dysfunction and remodeling by suppressing RAS parameters, oxidative stress, inflammation, and restoring the AT1R/PKC/NOX2/Raf‐1/ERK1/2 signaling pathway. These findings highlight naringin as a promising alternative for hypertension management.

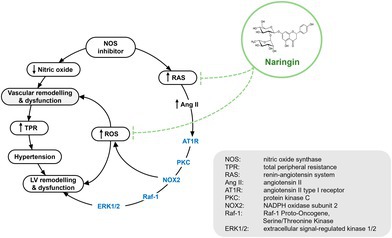

## 1 Introduction

**FIGURE 1:**
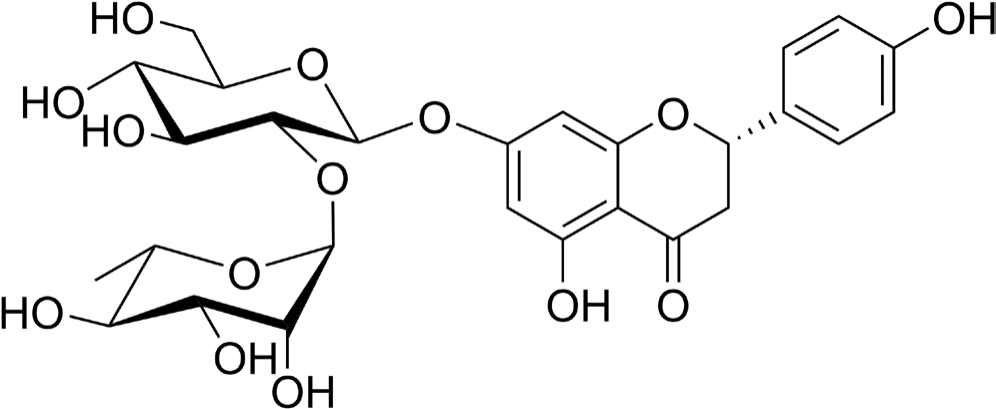
Structure of naringin (Chen et al. 2016).

Naringin, a flavonoid glycoside, is the main bioactive compound in citrus fruits (Zhang, Gao, et al. 2014). The molecular weight of naringin is 580.54 g/mol, and its chemical formula is C_27_H_32_O_14_. Naringin has a flavonoid structure because it comprises a heterocyclic pyran ring, two phenolic rings, and a disaccharide group connected at position 7 via a glycosidic bond (Figure 1). Naringin primarily imparts bitterness to citrus juice (Drewnowski et al. 1997). Several biological and pharmacological characteristics of naringin have been identified. For example, it exerts a potent antioxidant capacity to eliminate 1,1‐diphenyl‐2‐picrylhydrazyl (DPPH) free radical (Gerçek et al. 2021). Naringin has anti‐diabetic effects such as lowering blood glucose levels and oxidative stress parameters and restoring the circulating antioxidant defense system in diabetic rats (type 2) produced by streptozotocin (Mahmoud et al. 2012). Combined treatment of naringin and glimepiride produced a synergistic effect in alleviating metabolic parameters and kidney damage in rats with streptozotocin‐induced diabetes (Rath et al. 2023). Naringin exhibits anti‐inflammatory properties because it reduces interleukin‐1‐beta (IL‐1β), interleukin‐6 (IL‐6), and tumor necrosis factor‐alpha (TNF‐α) in rats with intestinal damage caused by ischemia/reperfusion (Gu et al. 2023). Furthermore, the antihypertensive effect of naringin has been documented in rats with diet‐induced metabolic syndrome and stroke‐prone spontaneously hypertensive rats (SHR) (Alam et al. 2013; Ikemura et al. 2012) and is associated with its antioxidative effect.

Hypertension, a chronic condition, is a significant contributor to the development of cardiovascular illnesses. An imbalance in the vasodilator/vasoconstrictor agents released from the endothelial cells can cause high blood pressure. The essential gas nitric oxide (NO), which is produced by vascular endothelial cells, controls blood pressure and vascular tone (Palmer et al. 1987). The continuous inhibition of NO production by NO synthase inhibitors induces arterial hypertension and organ damage in animals (Ribeiro et al. 1992). Blocking NO generation using *N*_ω_‐Nitro‐L‐arginine methyl ester hydrochloride (L‐NAME) causes rats to experience widespread narrowing of blood vessels and elevated blood pressure (Ribeiro et al. 1992). Decreased left ventricular (LV) function and reduced blood vessel relaxation because of endothelial dysfunction were observed in rats with NO deficiency (Kalra et al. 2020; Yang et al. 2019). Changes in the cardiovascular structure in rats, such as myocardial and aortic fibrosis, were observed because of blocking NO synthesis to generate hypertension (Chaihongsa et al. 2021; Kalra et al. 2020). Bernátová et al. found that chronic L‐NAME treatment induces myocardial fibrosis and aortic wall thickening in rats (Bernátová et al. 2002).

Several variables contribute to the development of cardiovascular dysfunction and remodeling in hypertensive rats. In L‐NAME‐treated rats, activation of the renin‐angiotensin system (RAS) maintains hypertension and mediates cardiovascular hypertrophy (On‐Nom et al. 2022; Zanchi et al. 1995). Sonoda et al. showed that chronic treatment with an L‐arginine analogue in rats can produce cardiac remodeling associated with angiotensin II (Ang II) downstream (Sonoda et al. 2017). Furthermore, Ang II increases angiotensin II type 1 receptor (AT1R)‐mediated generation of reactive oxygen species (ROS) and inflammatory cytokines (Griendling et al. 1997; Rincón et al. 2015). In L‐NAME‐treated rats, Ang II can trigger nicotinamide adenine dinucleotide phosphate (NADPH) oxidase (NOXs) to generate ROS, especially superoxide (Rincón et al. 2015). NOX2 and gp91^phox^ are mainly expressed in the plasma membranes of cardiomyocytes and are regulated by stretching or Ang II (Santillo et al. 2015). NOX2 facilitates ROS production and activates the Raf‐1/extracellular signal‐regulated kinases 1/2 (ERK1/2) signaling pathway to mediate myocardial fibrosis (Adamcova et al. 2021). In hypertensive rats, the pathophysiology of tissue fibrosis has been influenced by the inflammatory response, which is linked to a signaling cascade including cytokines and TNF‐α.

Currently, several antihypertensive drugs are used to control blood pressure. However, side effects and resistance to treatment can occur in patients with hypertension. Emerging evidence suggests that naringin can be used as an alternative or complementary treatment. However, the mechanism by which naringin prevents L‐NAME‐induced cardiovascular disturbances has not yet been explored. This study aimed to examine the protective effect of naringin against L‐NAME‐induced hypertension, cardiovascular dysfunction, and remodeling in rats, with particular emphasis on the activity of naringin in the RAS pathway as the underlying mechanism.

## 2 Methods

### 2.1 Chemicals

Naringin (product No. 71162) and L‐NAME (product No. N5751) were purchased from Sigma‐Aldrich (St. Louis, MO, USA). Telmisartan (batch No. E24966) was purchased from Boehringer Ingelheim Pharmaceutical (Ingelheim, Germany). All chemicals and reagents used in this study were purchased from trustworthy vendors, were of high quality, and were suitable for analysis.

### 2.2 Animals

The study utilized Male Sprague–Dawley rats (weighing 200–220 g) obtained from Nomura Siam International Co. Ltd., Bangkok, Thailand. The study was performed at the Northeast Laboratory Animal Center. All animal procedures were approved by the Animal Ethics Committee of Khon Kaen University, Khon Kaen, Thailand (IACUC‐KKU‐38/65), and adhered to the guidelines for the care and use of experimental animals. The rats were kept in a controlled environment with a heating, ventilation, and air‐conditioning system (maintained at a temperature of 23°C ± 2°C) and a 12 h cycle of darkness and light, and were given *ad libitum* access to food and water. The standard chow diet containing 5.72 g fat/100 g, 22.9 g protein/100 g, and 57.81 g carbohydrates/100 g was used in this study. The acclimatization period was 1 week before the experiment. Male Sprague–Dawley rats were given drinking water with 40 mg/kg body weight (BW)/day L‐NAME to induce hypertension and cardiovascular abnormalities, whereas normotensive rats received drinking water for a period of 5 weeks.

### 2.3 Experimental Protocols

Four rats were raised in the same cage, and 120 mL of L‐NAME solution was administered at an adjusted dose of 40 mg/kg BW/day. Following L‐NAME treatment, the rats were given regular drinking water for the rest of the day. The rats were intragastrically administered either vehicle (propylene glycol +5% dimethyl sulfoxide), naringin, or telmisartan daily for 5 weeks of the experimental period. In addition, unlimited food and water were provided to all rodents for the 5 weeks of treatment. The details of the animal groups and treatments are described below.

1. Control group (CG): vehicle (1.5 mL/kg BW/day)
2. Hypertensive group (HG): L‐NAME + vehicle (1.5 mL/kg BW/day)
3. HG + NR20: L‐NAME + naringin (20 mg/kg BW/day)
4. HG + NR40: L‐NAME + naringin (40 mg/kg BW/day)
5. HG + Tel: L‐NAME + telmisartan (5 mg/kg BW/day)

The dosage selection of naringin and telmisartan was guided by the preliminary study and a previous study (Cui et al. 2018; Xu and Liu 2013). In L‐NAME rats, we discovered that treatment with naringin at a dose of 40 mg/kg/day for 5 weeks had a higher effect on lowering blood pressure than treatment with a lower dose (20 mg/kg/day) of naringin (119.5 ± 10.66 vs. 167.7 ± 4.35 mmHg (mean ± SD, *n* = 3, *p* < 0.0019)). Naringin at doses of 20 and 40 mg/kg/day was thus investigated in this study.

### 2.4 Determination of Blood Pressure

A non‐invasive technique was applied to detect the systolic blood pressure (SBP) of conscious rats from the rat tail artery once a week using tail‐cuff plethysmography (CODA software, Kent Scientific., Torrington, CT, USA). Before blood pressure was measured, all rats were trained daily for a week to become accustomed to the restrainer and the procedure. Average SBP was calculated from three recordings for each rat.

### 2.5 Evaluation of Cardiac Function

Following 5 weeks of therapy, all rats underwent echocardiography using a GE Logiq S7 Ultrasound Machine (GE Healthcare, WI, USA) to determine heart function. The rats were administered an intraperitoneal dose of 60 mg/kg BW of thiopental sodium to induce anesthesia. The chest was cleaned and shaved to accommodate the probe. LV fractional shortening (LVFS) and ejection fraction (LVEF) were evaluated. Subsequently, the abdominal aorta was used to draw blood for biochemical testing.

### 2.6 Assessment of Vascular Function

The rats were exsanguinated and sacrificed. The thoracic aorta was carefully collected and divided into 0.3 cm sections to evaluate the vasoactive performance of the aortic rings. The aortic ring was immersed in a separate bath filled with 15 mL of Krebs solution at 37°C (gassed with carbogen). The fluctuations in isometric contraction were recorded using a transducer connected to a 4‐channel bridge amplifier and a PowerLab A/D converter (PowerLab System, ADInstruments, New South Wales, Australia) at a resting tension of 1 g. Before inducing vasodilation, the aortic rings were pre‐constricted with phenylephrine, an α1‐adrenergic receptor agonist, at a concentration of 10 μM. Then, acetylcholine (ACh), an endothelium‐dependent vasodilator, was added at concentrations ranging from 0.01 to 3 μM. Additionally, sodium nitroprusside (SNP), a nitric oxide donor, was added at concentrations ranging from 0.01 to 3 μM to produce vasodilation. The relaxation response was quantified as the percentage of contraction induced by phenylephrine.

### 2.7 Histological Studies

Thoracic aorta and LV tissues were preserved for 24 h in 4% paraformaldehyde. All tissues were paraffin‐embedded, sectioned into sequential 5 μm thick pieces, and stained with Picrosirius red (Polysciences, Warrington, PA, USA) to visualize the deposition of collagen. Images were obtained using a Nikon DS‐2Mv light microscope and an Eclipse Ci‐POL polarized light microscope (Tokyo, Japan). Morphometric evaluations and fibrosis levels were analyzed using image analysis software (ImageJ, National Institutes of Health, Bethesda, MD, USA). The proportion of the area fraction was used to express the collagen deposition in the heart and aorta.

### 2.8 Assay of Renin‐Angiotensin Parameters

Angiotensin converting enzyme (ACE) activity was measured in the serum using a previously described fluorescence assay (Maneesai et al. 2017). Hippuryl‐l‐histidyl‐l‐leucine and serum were combined in an assay buffer. The mixture was incubated at a constant temperature of 37°C for 30 min. O‐Phthaldialdehyde was used to label the product fluorogenically before NaOH was applied to stop the reaction. Fluorescence was measured using a fluorescence plate reader at excitation and emission wavelengths of 355 and 535 nm, respectively. ACE activity was described in mU/mL. An Ang II Enzyme Immunoassay kit (RAB0010‐1KT; St. Louis, MO, USA) was used to measure plasma Ang II concentrations. The protocol was consistent with the kit's procedure guidelines.

### 2.9 Assessment of Oxidative Stress Biomarkers and Enzymatic Antioxidants

#### 2.9.1 Formation of Superoxide in the Aortic Tissue

According to earlier descriptions (Maneesai et al. 2017), lucigenin‐enhanced chemiluminescence was used to assess superoxide formation in the aortic tissue. The aorta was rapidly removed and submerged in ice‐cold saline to remove the fat and connective tissue. The tissue was cut into 2 cm lengths and incubated for 30 min at 37°C in Krebs KCl buffer with a pH of 7.4. Subsequently, the sample tube was filled with lucigenin and placed in a luminometer (Turner Biosystems, Sunnyvale, CA, USA), which recorded integration every 30 s for 5 min. The tissue was then dried at 45°C for 24 h to ascertain its dry weight. The relative light unit count per mg of dry tissue weight per min represents superoxide generation in the tissue and was used to express the results.

#### 2.9.2 Plasma Malondialdehyde (MDA)

The concentration of thiobarbituric acid‐reactive compounds was used to indirectly quantify the concentration of MDA in the plasma (Bunbupha et al. 2015). Next, 10% trichloroacetic acid, 5 mmol/L ethylenediaminetetraacetic acid, 8% sodium dodecyl sulfate, and 0.5 μg/mL butylated hydroxytoluene was added in 150 μL of the plasma. After 10 min of incubation at 24°C, the mixture was boiled in a water bath for 30 min, followed by the addition of thiobarbituric acid (0.6%). After cooling to ambient temperature, the mixture was centrifuged at 10,000× *g* for 5 min. The absorbance of each sample was measured at 532 nm using an Amersham spectrophotometer (Arlington, MA, USA). A calibration curve was constructed using 1,1,3,3‐tetraethoxypropane at a concentration of 0.3–10 μmol/L.

#### 2.9.3 Catalase Activity

Plasma catalase (CAT) enzyme activity was quantified using a modified version of a previously established method (Góth 1991; Özmen et al. 2002). In brief, samples were incubated with the substrate (65 μmol/mL of H_2_O_2_ in 60 mmol/L sodium‐potassium phosphate buffer pH 7.4) in a 96‐well plate at 37°C for 1 min. Next, 32.4 mmol/L ammonium molybdate was added to terminate the reaction. The absorbance of the yellowish molybdate and H_2_O_2_ complex was determined at 405 nm to calculate the CAT activity level.

#### 2.9.4 Superoxide Dismutase (SOD) Activity

A SOD assay kit (Sigma‐Aldrich, St. Louis, MO, USA) was used, and SOD activity was colorimetrically analyzed using a spectrophotometer. Briefly, 20 μL of the sample solution was added to each sample and blank, followed by the addition of 20 μL of ultrapure water to Blanks 1 and 3. Next, 200 μL of WST working solution was added to each well and stirred. Dilution buffer (20 μL) was added to the wells of Blanks 2 and 3. Subsequently, 20 μL of enzyme working solution was added to each sample and Blank 1, mixed, and incubated at 37°C for 20 min. The absorbance was measured at 450 nm using a microplate reader (Tecan, Grodig, Austria). The SOD activity was calculated as the percentage of inhibition.

#### 2.9.5 Nitrate/Nitrite Concentration

Plasma nitrate/nitrite concentrations were quantified using an enzymatic conversion technique with certain modifications (Nakmareong et al. 2011). In summary, plasma samples were purified by removing proteins by ultrafiltration using centrifugal concentrators (Pall Corp., Ann Arbor, MI, USA). The liquid portion was combined with a solution containing 1.2 μmol/L of NADPH, 4 mmol/L of glucose‐6‐phosphate disodium, 1.28 U/mL of glucose‐6‐phosphate dehydrogenase, and 0.2 U/mL of nitrate reductase. Subsequently, the mixture was maintained at a temperature of 30°C for a duration of 30 min. The mixture was then mixed with the Griess solution, which contained 4% sulfanilamide in 0.3% N‐(1‐napthyl) ethylenediamine dihydrochloride, and left to react for 15 min. Absorbance was measured at 540 nm using a microplate reader (Tecan, Grodig, Austria).

### 2.10 Assessment of Plasma TNF‐α

The quantity of TNF‐α in plasma was determined using an enzyme immunoassay test (RAB0479; Sigma‐Aldrich Inc., Saint Louis, USA). Briefly, samples or standard solution were added to 96 wells, covered, and incubated at 24°C for 2.5 h with gentle shaking. The solution was discarded and washed four times with 1× wash solution. Subsequently, biotinylated anti‐rat TNF antibody was added and incubated for 1 h at room temperature with gentle shaking. The wells were washed again before adding HRP‐conjugated streptavidin. After washing the wells, a TMB substrate solution was added and incubated for 30 min at room temperature with gentle shaking in the dark. Thereafter, stop solution was added, and the color intensity was immediately measured at 450 nm.

### 2.11 Protein Expression Analysis

The western blot method was employed to detect the expression of AT1R, protein kinase C‐alpha (PKC‐α), gp91phox, Raf‐1, and ERK1/2 protein in the cardiac tissue. Protein concentration was detected by Bradford protein assay. Following the homogenization of the heart tissues, 20 μg of the homogenate proteins was separated using sodium dodecyl sulfate‐polyacrylamide gel electrophoresis. Subsequently, proteins were transferred onto a polyvinylidene difluoride membrane and then blocked for 2 h at room temperature using a solution of 5% skim milk in Tris‐buffered saline containing 0.1% Tween 20. Next, the sample was incubated overnight at 4°C using mouse monoclonal antibodies specific to the following targets: AT1R (G‐3) (sc‐515884), gp91phox (G‐1) mouse monoclonal IgG (sc‐74514), Rb mAb to PKC‐α (Y124) (ab32376), Rb pAb to Raf‐1 (ab137435), and Rb pAb to ERK1 + ERK2 (ab17942) (Abcam, Plc, Cambridge, UK). Subsequently, the membranes were washed and exposed to a secondary antibody conjugated with horseradish peroxidase for 2 h at ambient temperature. Densitometric analysis was conducted using the ImageQuant 400 system (GE Healthcare Life Sciences, Piscataway, NJ, USA), following the development of blots in the Amersham ECLTM Prime solution (Amersham Biosciences Corp., Piscataway, NJ, USA). The β‐actin (C4) mouse monoclonal IgG (sc‐47778; Santa Cruz Biotechnology Inc., Dallas, TX, USA) was used as the reference to calculate all bands intensities. Data were presented as the percentage of values found on the identical gel in the control group.

### 2.12 Statistical Analysis

All data are presented as the mean ± standard deviation (SD). The SBP data were tested using a two‐way analysis of variance (ANOVA). When significant differences (*p* < 0.05) were discovered, a Tukey's post hoc analysis was performed. Other data were examined using one‐way ANOVA, which was followed by Tukey's post hoc test to determine group differences. Statistical significance was set at *p* < 0.05. Data were found to be normally distributed as indicated by the Shapiro–Wilk test. Data were analyzed using GraphPad Prism 9.5.

## 3 Results

### 3.1 Naringin Prevents Hypertension in L‐NAME‐Treated Rats

**FIGURE 2:**
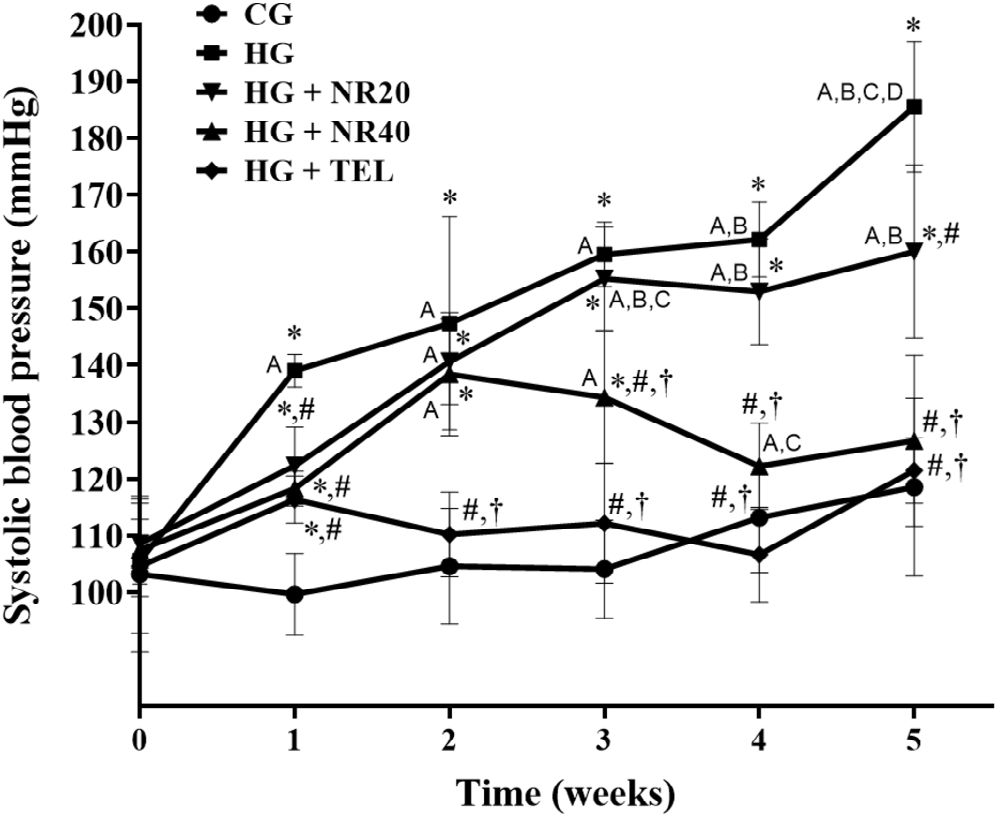
Effects of naringin or telmisartan on systolic blood pressure in the HG rats. Data are expressed as mean ± SD (*n* = 8). *p < 0.05 vs. CG, ^#^ *p* < 0.05 vs. HG, ^†^ *p* < 0.05 vs. HG + NR20. ^A^ *p* < 0.05 vs. week 0, ^B^ *p* < 0.05 vs. week 1, ^C^ *p* < 0.05 vs. week 2, ^D^ *p* < 0.05 vs. week 4. Data were analyzed using a two‐way ANOVA, followed by Tukey's post hoc test. CG, control group; HG, hypertensive group; NR20, naringin (20 mg/kg BW/day); NR40, naringin (40 mg/kg BW/day); TEL, telmisartan (5 mg/kg BW/day).

The basal SBP of the experimental groups did not exhibit any significant difference. SBP was substantially elevated in the HG (185.55 ± 11.53 mmHg) compared to the CG (118.56 ± 15.68 mmHg) after 5 weeks of administration (*p* < 0.0001). The gradual increase in blood pressure induced by L‐NAME was reduced in rats treated with naringin (NR20; 160.06 ± 15.26 mmHg, *p* = 0.02, NR40; 126.73 ± 15.10 mmHg, *p* < 0.0001 and telmisartan; 121.48 ± 5.76 mmHg, *p* < 0.0001) compared to HG. Moreover, a higher dose of naringin (40 mg/kg BW/day) prevented L‐NAME‐induced hypertension more effectively than a lower dose (20 mg/kg BW/day) (*p* = 0.005) (Figure 2). SBP of HG and HG + NR20 at 5 weeks was significantly higher than the value at the beginning of the experiment (HG; 105.58 ± 4.15 mmHg (*p* = 0.0003), NR20; 105.62 ± 4.28 mmHg, *p* = 0.02).

### 3.2 Organ Weight in All Rats

CG, HG, HG + NR20, HG + NR40, and HG + TEL exhibited no discernible disparities in BW after 5 weeks of therapy. However, the HG exhibited substantially higher heart weight (HW)/BW (*p* = 0.007) ventricular weights (VW)/BW (*p* = 0.03), and left VW (LVW)/BW (*p* = 0.01) ratios than the CG (*p* < 0.05; Table 1). Naringin (40 mg/kg BW/day) and telmisartan markedly decreased the HW/BW (*p* < 0.0001 and *p* = 0.0003), VW/BW (*p* = 0.0005 and *p* < 0.0001), and LVW/BW (*p* = 0.002 and *p* = 0.02) ratios compared to the untreated HG (Table 1). In addition, naringin (40 mg/kg BW/day) reduced HW/BW (*p* = 0.002) and LVW/BW (*p* = 0.02) ratios more effectively than a lower dose in hypertensive rats.

**TABLE 1:** Body and heart weights of rats in all the groups.

Parameters	CG	HG	HG + NR20	HG + NR40	HG + TEL
BW (g)	462.69 ± 22.88	453.99 ± 16.96	454.60 ± 23.81	453.84 ± 16.28	432.83 ± 18.48
HW/BW (mg/g BW)	3.03 ± 0.22	3.31 ± 0.27*	3.06 ± 0.11	2.90 ± 0.13^#,†^	2.81 ± 0.09^#^
VW/BW (mg/g BW)	2.42 ± 0.06	2.72 ± 0.22*	2.57 ± 0.10	2.32 ± 0.15^#^	2.34 ± 0.14^#^
LVW/BW (mg/g BW)	1.88 ± 0.07	2.11 ± 0.16*	2.06 ± 0.12*	1.84 ± 0.06^#,†^	1.90 ± 0.19^#^

*Note:* Data are expressed as mean ± SD (*n* = 8). *p < 0.05 vs. CG, ^#^ *p* < 0.05 vs. HG, ^†^ *p* < 0.05 vs. HG + NR20. Differences among groups were analyzed using one‐way analysis of variance, followed by Tukey's post hoc test.

Abbreviations: BW, body weight; CG, control group; HG, hypertensive group; HW/BW, heart weight/body weight ratio; LVW/BW, left ventricular weight/body weight ratio; NR20, naringin (20 mg/kg BW/day); NR40, naringin (40 mg/kg BW/day); TEL, telmisartan (5 mg/kg BW/day); VW/BW, ventricular weight/body weight ratio.

### 3.3 Naringin Preserves Cardiac Performance in Hypertensive Rats

**FIGURE 3:**
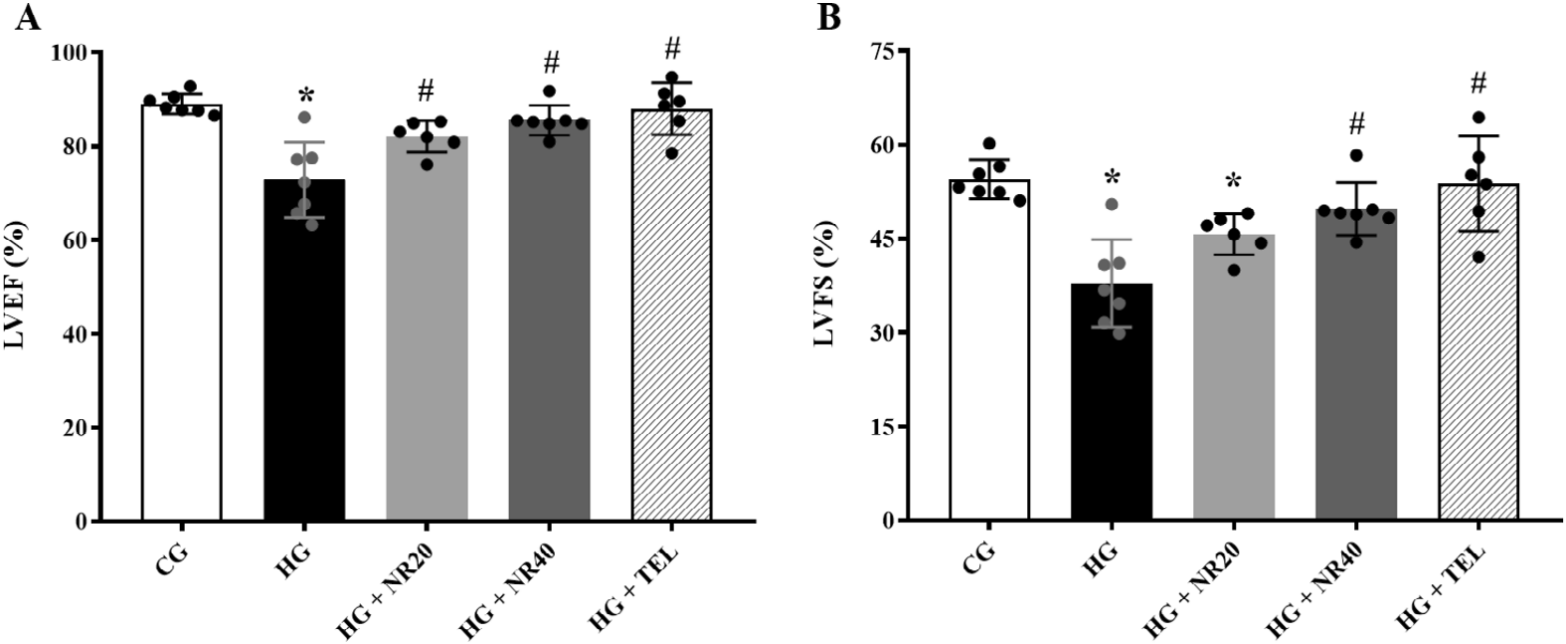
Effects of naringin or telmisartan on (A) % LVEF and (B) % LVFS in the HG. Data are expressed as mean ± SD (*n* = 6–7). *p < 0.05 vs. CG, ^#^ *p* < 0.05 vs. HG. Differences among groups were analyzed using one‐way analysis of variance, followed by Tukey's post hoc test. CG, control group; HG, hypertensive group; LVEF, left ventricular ejection fraction; LVFS, left ventricular fractional shortening; NR20, naringin (20 mg/kg BW/day); NR40, naringin (40 mg/kg BW/day); TEL, telmisartan (5 mg/kg BW/day).

Rats administered L‐NAME exhibited a substantial decrease in both LVEF (72.81 ± 8.05, *p* < 0.0001) and LVFS (37.91 ± 6.99, *p* < 0.0001) compared to CG (LVEF; 89.00 ± 2.12 and LEFS; 54.50 ± 3.13). As shown in Figure 3, cardiac function was improved in hypertensive rats treated with telmisartan (LVEF; 88.02 ± 5.57, *p* < 0.0001 and LVFS; 53.80 ± 7.59, *p* < 0.0001), naringin (20 mg/kg BW/day) (LVEF; 82.03 ± 3.36, *p* = 0.02) or naringin (40 mg/kg BW/day) (LVEF; 85.50 ± 3.18, *p* = 0.0004; LVFS; 49.75 ± 4.18, *p* = 0.002) compared to HG.

### 3.4 Naringin Improves Endothelial Function in the Aortic Rings

**FIGURE 4:**
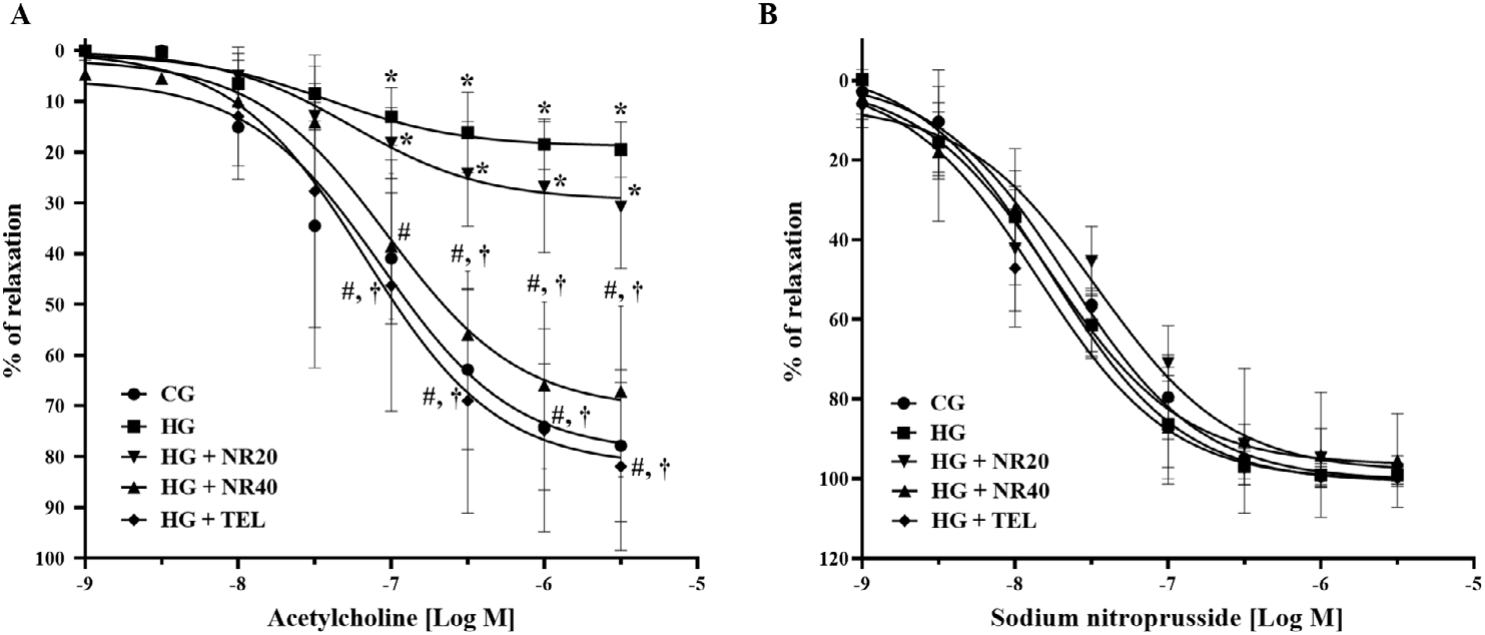
Effects of naringin or telmisartan on the vascular response to (A) acetylcholine and (B) sodium nitroprusside in aortic rings. Data are expressed as mean ± SD (*n* = 8). *p < 0.05 vs. CG, ^#^ *p* < 0.05 vs. HG, ^†^ *p* < 0.05 vs. HG + NR20. Differences among groups were analyzed using one‐way analysis of variance, followed by the Tukey's post hoc test. CG, control group; HG, hypertensive group; NR20, naringin (20 mg/kg BW/day); NR40, naringin (40 mg/kg BW/day); TEL, telmisartan (5 mg/kg BW/day).

Compared with the control rats, aortic rings of the HG exhibited a considerably lower vascular response to ACh (the highest dose; 77.82 ± 14.93 vs. 19.46 ± 5.40, *p* < 0.0001). Compared to the untreated group, the HG that received naringin or telmisartan demonstrated a substantial improvement in endothelium‐dependent vasorelaxation (NR20; 30.73 ± 12.12, NR40; 67.11 ± 16.76, TEL; 81.88 ± 16.49, *p* < 0.0001) (Figure 4A). The vasorelaxation response to ACh in HG + NR20 was less than HG + NR40 (*p* = 0.0009) and HG + TEL (*p* < 0.0001). A concentration‐dependent vasodilatory response to SNP was observed, but no significant difference between the groups was found (Figure 4B).

### 3.5 Naringin Alleviates Cardiac Fibrosis

**FIGURE 5:**
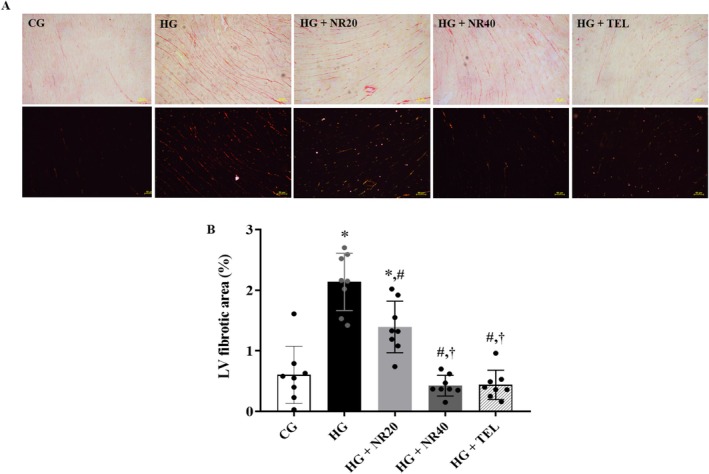
(A) Representative image of myocardial fibrosis under light and polarized light microscopes, scale bar: 50 μm. (B) Effects of naringin or telmisartan on the left ventricular fibrotic area in hypertensive rats. Data are expressed as mean ± SD (*n* = 8). *p < 0.05 vs. CG, ^#^ *p* < 0.05 vs. HG, ^†^ *p* < 0.05 vs. HG + NR20. Differences among groups were analyzed using one‐way analysis of variance, followed by the Tukey's post hoc test. CG, control group; HG, hypertensive group; NR20, naringin (20 mg/kg BW/day); NR40, naringin (40 mg/kg BW/day); TEL, telmisartan (5 mg/kg BW/day).

Compared with the control rats, the LV fibrotic area was larger in the HG that received vehicle (*p* < 0.0001). Heart fibrosis caused by hypertension in rats was reduced after 5 weeks of naringin (20 and 40 mg/kg BW/day) or telmisartan treatment, as shown in Figure 5 (*p* = 0.003, *p* < 0.0001 and *p* < 0.0001, respectively) compared to HG. Furthermore, naringin at a dose of 40 mg/kg BW/day produced a greater effect on lowering fibrotic area than naringin at a dose of 20 mg/kg BW/day (*p* = 0.0001).

### 3.6 Naringin Alleviates Aortic Hypertrophy and Fibrotic Area

**FIGURE 6:**
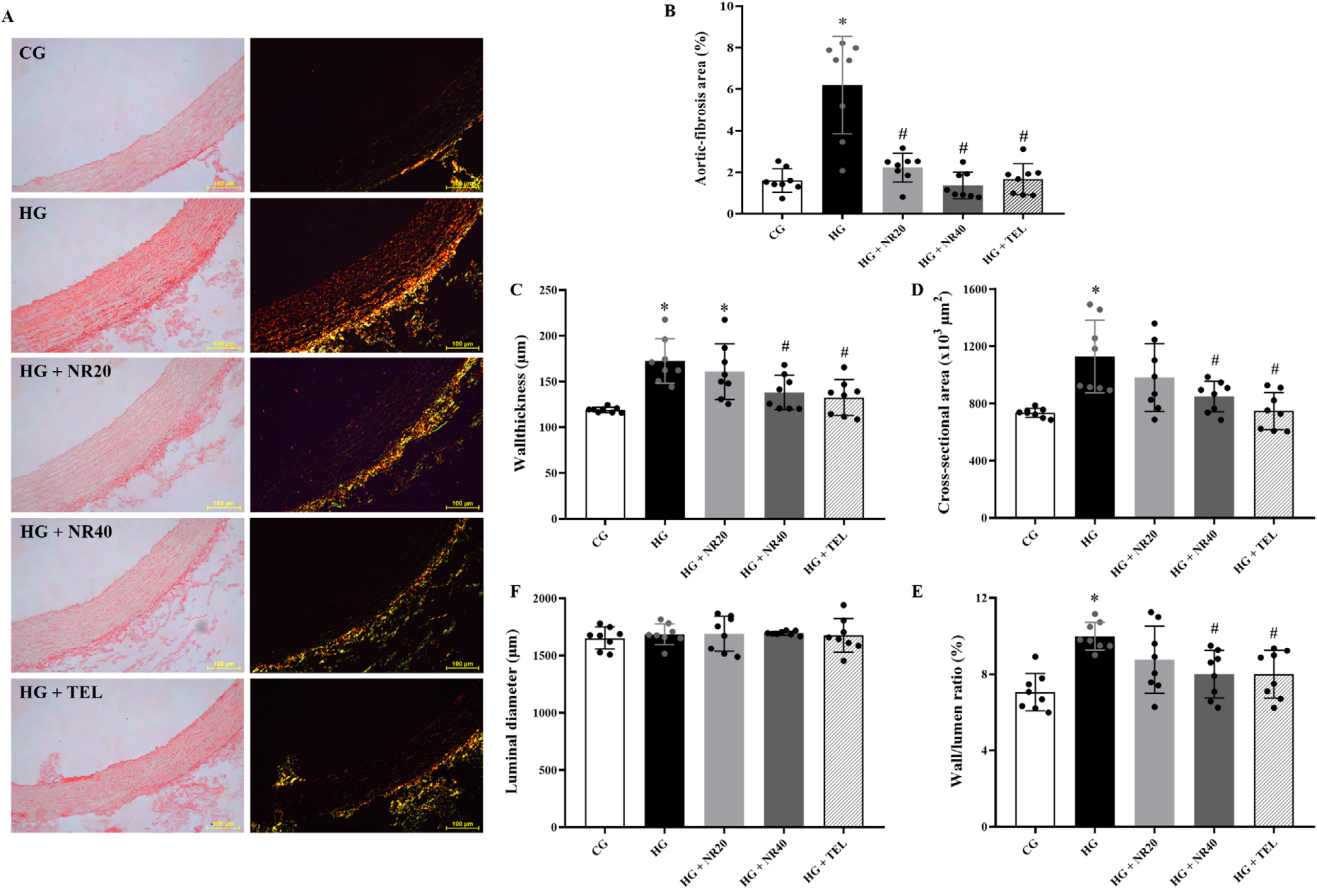
(A) Representative image of aortic fibrosis under light and polarized light microscopes in all experimental groups, scale bar: 100 μm. Effect of naringin or telmisartan on (B) aortic‐fibrotic area, (C) wall thickness, (D) cross‐sectional area, (E) luminal area, and (F) wall/lm ratio in all groups. Data are expressed as mean ± SD (*n* = 8). *p < 0.05 vs. CG, ^#^ *p* < 0.05 vs. HG. Differences among groups were analyzed using one‐way analysis of variance, followed by the Tukey's post hoc test. CG, control group; HG, hypertensive group; NR20, naringin (20 mg/kg BW/day); NR40, naringin (40 mg/kg BW/day); TEL, telmisartan (5 mg/kg BW/day).

The aorta of the L‐NAME animals exhibited collagen accumulation in comparison to that of the CG, as evidenced by picrosirius red staining (Figure 6A,B; *p* < 0.0001). The HG exhibited an increase in the aortic wall thickness, cross‐sectional area, and wall‐to‐lumen ratio (Figure 6C,D,F; *p* < 0.0001, *p* = 0.0006, *p* = 0.0003, respectively) compared to CG. However, the aortic luminal diameter did not differ between the groups (Figure 6E). Daily supplementation of telmisartan or naringin (20 mg/kg BW/day) or naringin (40 mg/kg BW/day) decreased the aortic fibrotic area in the L‐NAME‐treated group compared to that in the untreated group (Figure 6A,B, *p* < 0.0001). Furthermore, 40 mg/kg BW/day naringin or telmisartan treatment reversed aortic hypertrophy, including the aortic wall thickness (*p* = 0.02 or *p* = 0.005), cross‐sectional area (*p* = 0.02 or *p* = 0.0009), and wall‐to‐lumen ratio (*p* = 0.02 or *p* = 0.02) compared to the HG (Figure 6C,D,F; *p* < 0.05).

### 3.7 Naringin Suppressed Renin‐Angiotensin System Parameters

**FIGURE 7:**
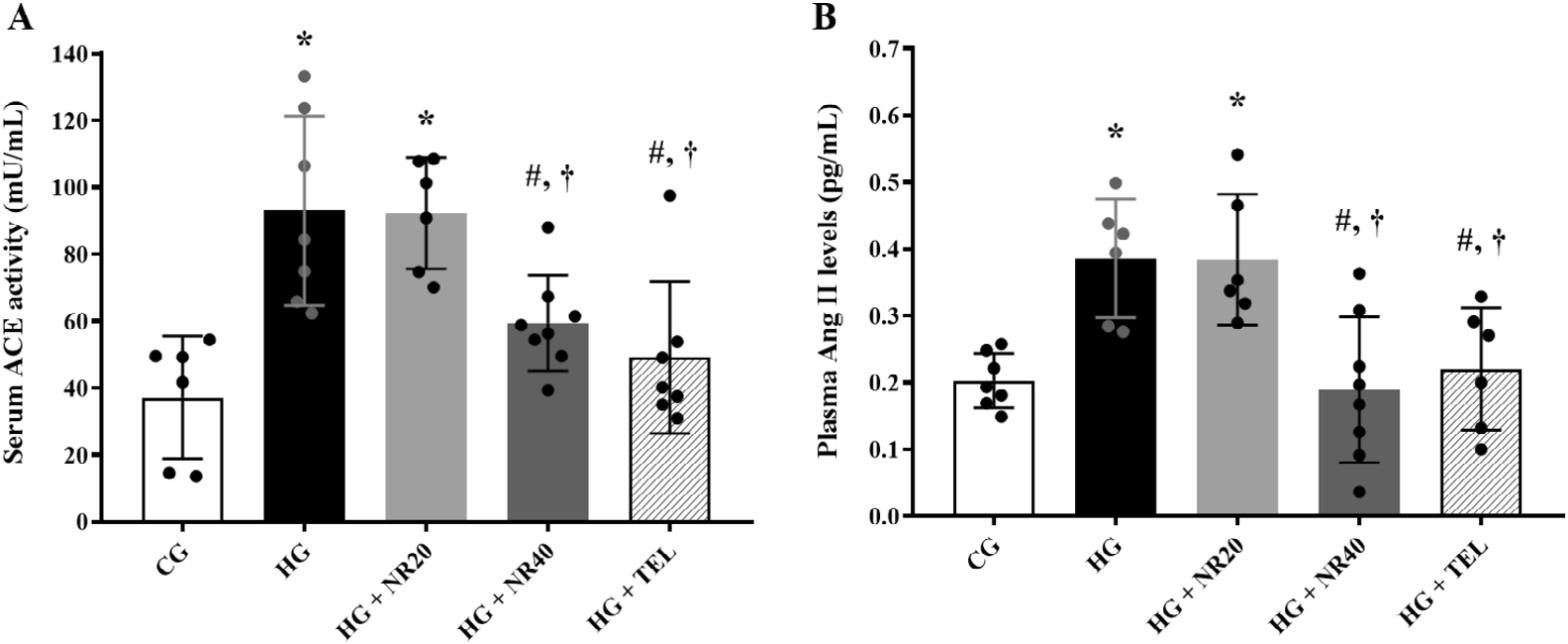
Effects of naringin or telmisartan on (A) serum ACE activity and (B) plasma Ang II levels in hypertensive rats. Data are expressed as mean ± SD (*n* = 6–8). *p < 0.05 vs. CG, ^#^ *p* < 0.05 vs. HG, ^†^ *p* < 0.05 vs. HG + NR20. The differences among groups were analyzed using one‐way analysis of variance, followed by the Tukey's post hoc test. ACE, angiotensin‐converting enzyme; Ang II, angiotensin II; CG, control group; HG, hypertensive group; NR20, naringin (20 mg/kg BW/day); NR40, naringin (40 mg/kg BW/day); TEL, telmisartan (5 mg/kg BW/day).

Compared with the CG, the HG displayed RAS activation accompanied by elevated concentrations of circulating ACE (37.20 ± 18.36 vs. 93.03 ± 28.36, *p* = 0.0004) activity and Ang II (0.20 ± 0.04 vs. 0.39 ± 0.09, *p* = 0.008; Figure 7A,B). Additionally, as seen in Figure 7A,B, the HG that received 40 mg/kg BW/day of naringin (ACE; 63.74 ± 18.57, *p* = 0.03 and Ang II; 0.19 ± 0.11, *p* = 0.003) or telmisartan (ACE; 49.17 ± 22.76, *p* = 0.004 and Ang II; 0.19 ± 0.12, *p* = 0.03) showed restoration of RAS parameters in comparison to the untreated HG.

### 3.8 Effects of Naringin on Oxidative Stress and Inflammatory Markers

The HG showed significantly lower plasma concentrations of nitrate/nitrite (*p* < 0.0001), SOD activity (*p* < 0.0001) and catalase (*p* = 0.03) as well as significantly higher concentrations of MDA (*p* < 0.0001) and aortic superoxide production (*p* < 0.0001) than those of the CG. Naringin (40 mg/kg BW/day) restored circulating nitrate/nitrite (*p* = 0.02), SOD activity (*p* = 0.0004), catalase (*p* = 0.04), MDA (*p* < 0.0001), and aortic superoxide production (*p* < 0.0001) compared to untreated hypertensive rats. Telmisartan normalized SOD activity (*p* = 0.001), MDA (*p* < 0.0001), aortic superoxide production (*p* < 0.0001), and circulating nitrate/nitrite (*p* = 0.03) compared to untreated hypertensive rats. Moreover, the HG had higher plasma TNF‐α concentrations than the CG (*p* = 0.002). HG plus 40 mg/kg BW/day of naringin (*p* = 0.01), or telmisartan (*p* = 0.03) restored the degree of inflammatory indicators compared to untreated HG (Table 2).

**TABLE 2:** Oxidative stress and inflammatory markers in hypertensive rats.

Parameters	CG	HG	HG + NR20	HG + NR40	HG + TEL
Plasma MDA levels (μM)	3.20 ± 0.64	7.91 ± 0.93*	6.71 ± 0.6*	4.33 ± 0.66^#,†^	3.11 ± 0.59^#,†^
Vascular superoxide production (counts/mg dry weight/min)	29.65 ± 12.69	115.56 ± 16.19*	100.48 ± 34.49*	51.23 ± 8.69^#,†^	52.34 ± 23.17^#,†^
Plasma CAT activity (U/mL)	257.94 ± 27.79	155.09 ± 58.32*	155.07 ± 83.73*	248.16 ± 17.97^#,†^	237.77 ± 24.49^#,†^
Plasma SOD activity (% inhibition rate)	99.21 ± 2.4	93.75 ± 5.21*	95.18 ± 2.62*	98.37 ± 0.68^#,†^	98.32 ± 0.78^#,†^
Plasma nitrate/nitrite (μM)	17.73 ± 4.08	10.77 ± 1.03*	12.28 ± 1.49 *	14.99 ± 0.97 ^#^	14.67 ± 2.18^#^
Plasma TNF‐α (pg/mL)	290.35 ± 239.49	902.71 ± 383.61*	523.33 ± 261.17*	389.60 ± 261.61^#^	437.78 ± 224.83^#^

*Note:* Data are expressed as mean ± SD (*n* = 8). *p < 0.05 vs. CG, ^#^ *p* < 0.05 vs. HG, ^†^ *p* < 0.05 vs. HG + NR20. Differences among groups were analyzed using one‐way analysis of variance, followed by Tukey's post hoc test.

Abbreviations: CAT, catalase; CG, control group; HG, hypertensive group; MDA, malondialdehyde; NR20, naringin (20 mg/kg BW/day); NR40, naringin (40 mg/kg BW/day); SOD, superoxide dismutase; TEL, telmisartan (5 mg/kg BW/day); TNF‐α, tumor necrosis factor‐alpha.

### 3.9 Effects of Naringin on the Expression of AT1R, PKC‐α, gp91^phox^, Raf‐1, and ERK1/2 Proteins on the Heart Tissue

**FIGURE 8:**
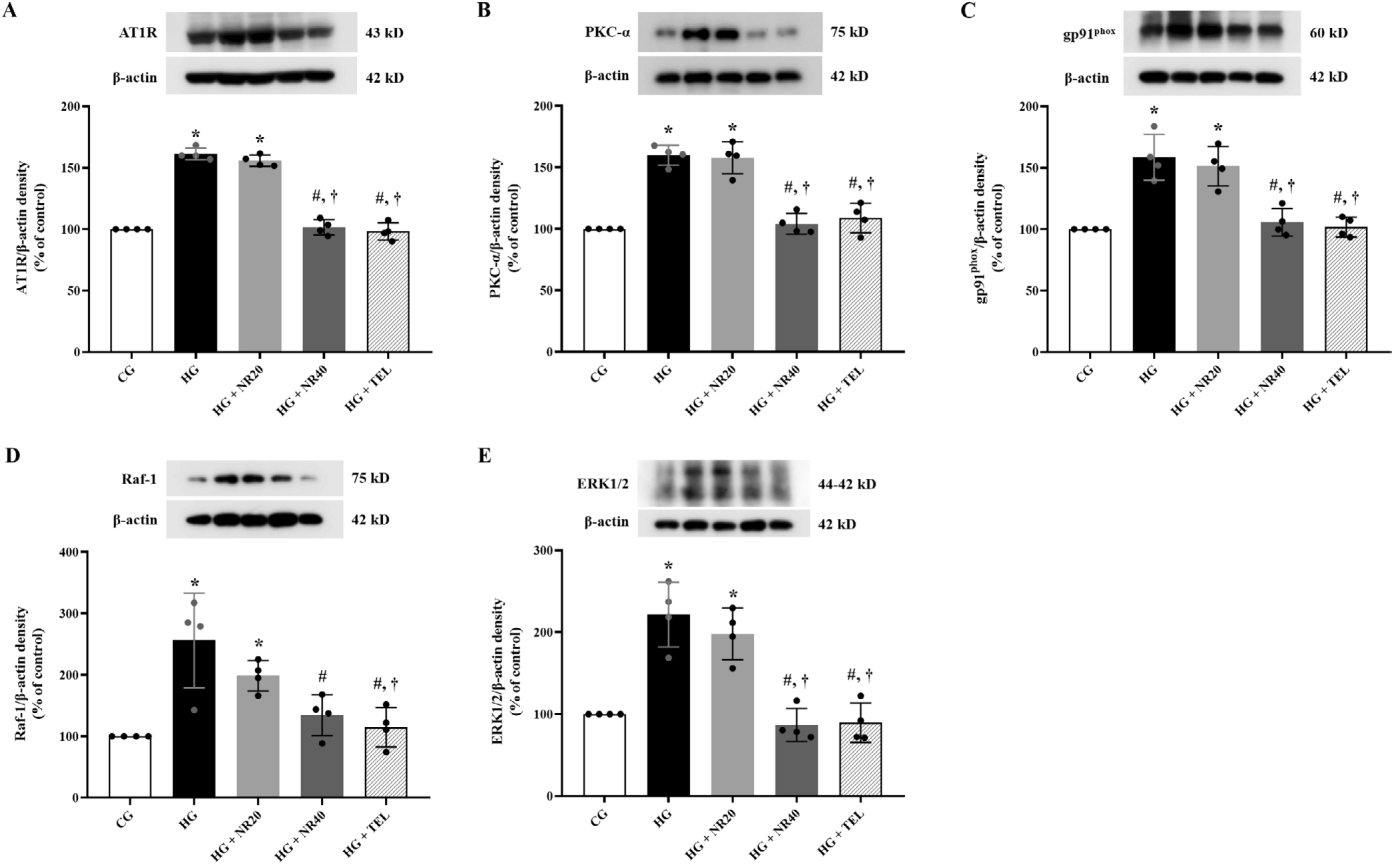
Effects of naringin or telmisartan on the expression of (A) angiotensin II type 1 receptor (AT1R), (B) protein kinase C‐alpha (PKC‐α), (C) gp91^phox^ (or NOX2), (D) Raf‐1, and (E) extracellular signal‐related kinases 1/2 (ERK1/2) proteins in the heart tissue. Data are expressed as mean ± SD (*n* = 4). *p < 0.05 vs. CG, ^#^ *p* < 0.05 vs. HG, ^†^ *p* < 0.05 vs. HG + NR20. Differences among groups were analyzed using a one‐way analysis of variance, followed by Tukey's post hoc test. CG, control group; HG, hypertensive group; NR20, naringin (20 mg/kg BW/day); NR40, naringin (40 mg/kg BW/day); TEL, telmisartan (5 mg/kg BW/day).

Western blot analysis of the cardiac tissue revealed that the HG had higher levels of PKC‐α (*p* < 0.0001), gp91^phox^ (*p* < 0.0001), Raf‐1 (*p* < 0.0001), and ERK1/2 (*p* < 0.0001) protein expression than the CG (Figure 8). Levels of the overexpression of these proteins were significantly reduced in the HG + NR40 (AT1R; *p* < 0.0001, PKC‐α; *p* < 0.0001, gp91^phox^; *p* = 0.0002, Raf‐1; *p* = 0.0001, and ERK1/2; *p* < 0.0001) and HG + TEL (AT1R; *p* < 0.0001, PKC‐α; *p* < 0.0001, gp91^phox^; *p* = 0.0001, Raf‐1; *p* < 0.0001, and ERK1/2, *p* < 0.0001) compared to those of the HG.

## 4 Discussion

In the present study, naringin partially protected rats against L‐NAME‐induced hypertension. Naringin supplementation reversed the decrease in LV function and endothelium‐dependent vasorelaxation in hypertensive rats. Moreover, it decreased the amount of collagen deposited in the aortic and cardiac tissues. Thickening of the aorta was observed in the HG and was alleviated by naringin treatment. In the HG, the protective action of naringin on the physiological and anatomical parameters of the cardiovascular system was associated with the inhibitory effects on ACE activity, Ang II content, oxidative stress, and inflammation. Naringin reduced AT1R/PKC/NOX2/Raf‐1/ERK1/2 expression in both LV tissues. Telmisartan was utilized as a positive control treatment in the current investigation and showed a cardioprotective effect comparable to naringin.

HG plus a high dose of naringin resulted in normal SBP values at the end of 5 weeks. Long‐term treatment with NO synthase inhibitor results in high blood pressure in animals (Ribeiro et al. 1992). In this study, naringin alleviated cardiovascular disturbances, including LV and endothelial dysfunction, in NO‐depleted rats. These results agree with those of other studies that used different models of hypertension, showing that naringin possesses antihypertensive and cardioprotective effects. The antihypertensive and anti‐thrombotic effects of naringin in stroke‐prone SHR are related to the restoration of endothelium‐dependent vasodilation and increased levels of NO metabolites in the urine (Ikemura et al. 2012). Furthermore, in rodents with renal artery occlusion, the antioxidant effect of naringin has been shown to enhance LV function and reduce blood pressure (Visnagri et al. 2015). L‐NAME‐induced hypertension, characterized by the enhancement of RAS, oxidative stress, and inflammatory factors, was investigated in this study. Hypertensive rats treated with naringin recovered their Ang II levels and ACE activity. Therefore, naringin likely exhibits ACE‐inhibitory effects, resulting in reduced Ang II levels in this animal model. Inhibition of ACE activity by flavanone‐like naringin was confirmed using a fluorometric method (Guerrero et al. 2012). A higher dose of naringin (40 mg/kg BW/day) decreased blood pressure, improved vascular function, and suppressed RAS more effectively than a lower dose (20 mg/kg BW/day). This result indicated that naringin at a dose of 40 mg/kg BW/day was effective.

Moreover, naringin reduced ROS and lipid peroxidation and enhanced SOD and CAT activity, which may contribute to its antihypertensive effect. The antioxidant properties of naringin were thought to improve endothelial function in this study. The lack of NO is a key factor in impairing endothelium‐dependent vasorelaxation and increasing the total peripheral resistance. Low levels of endogenous antioxidant enzymes and NO, along with elevated ROS levels, were frequently observed in the L‐NAME‐treated rats (Chia et al. 2021). In hypertensive animals, we found that naringin reduced oxidative damage and increased NO bioavailability. Our findings are supported by the observation that superoxide scavenging increased NO bioavailability and reduced the production of peroxynitrite radicals (Piacenza et al. 2022). In addition, naringin enhanced LV function in hypertensive rats. This observation is supported by a previous report showing that naringin normalizes LV function in high‐carbohydrate, high‐fat diet‐induced obese rats by reducing oxidative stress and inflammation (Alam et al. 2013). The reduction in LV performance in the HG results from LV remodeling. Therefore, in addition to oxidative stress, the molecular mechanism of naringin‐induced LV remodeling improved by naringin was elucidated in the present study. Moreover, naringin exhibited dose‐dependent antioxidant effects that were compatible with its effects on NO bioavailability and vascular function. The dose‐dependent effect of naringin on vascular function may be mediated by its effects on oxidative stress and NO.

LV hypertrophy and collagen deposition were supported by an increase in LVW/BW, and aortic fibrotic areas were observed in the HG. These morphological changes were reversed after naringin treatment. The mechanism of cardiac fibrosis in this study may involve the RAS, as Ang II promotes LV hypertrophy and remodeling via AT1R and its effector molecules (Sadoshima and Izumo 1993). Naringin modulates the Ang II/AT1R signaling pathway. It suppresses the overexpression of AT1R/PKC/NOX2/Raf‐1/ERK1/2 in L‐NAME‐treated rats. Ang II can activate ROS production via NOX2, which is highly expressed in the cardiac tissue (Looi et al. 2008). ROS activates the Raf‐2/ERK1/2 signaling pathway to induce myocardial fibrosis (Adamcova et al. 2021). Naringin also affects vascular remodeling by reducing aortic hypertrophy and fibrosis. In this study, the mechanism by which naringin alleviated vascular changes involved its anti‐inflammatory action. Oxidative stress and inflammatory processes have been proposed to be involved in the pathophysiology of vascular remodeling (Therrien et al. 2012). We found high levels of plasma TNF‐α and this abnormality was restored by naringin. Our results are consistent with those of many studies that have demonstrated the potential effect of naringin in reducing inflammation (Deenonpoe et al. 2019; Gu et al. 2023). Nevertheless, because the protein expression implicated in the inflammatory reactions in aortic tissue has not been assessed, this work has certain limitations.

We designed an experiment with a positive CG that used the standard antihypertensive drug telmisartan. High SBP and circulatory alterations induced by L‐NAME were prevented by telmisartan (5 mg/kg BW/day), an Ang II receptor blocker. Our results are supported by those of an earlier study that demonstrated that telmisartan lowers hypertension, cardiovascular remodeling, and oxidative stress in hypertensive rats sensitive to Dahl's salt (Kobayashi et al. 2008). In addition, telmisartan normalized LV function and fibrosis, which are associated with RAS antagonization in hypertensive rats (Zhang, Shao, et al. 2014). The protective mechanism of telmisartan against myocardial fibrosis after acute myocardial infarction is related to a reduction in inflammatory cytokines in rats (Song et al. 2014).

In conclusion, these results suggest that naringin exerts cardiovascular preventive effects induced by high blood pressure in rats. These effects are related to the inhibition of the RAS, oxidative stress, and inflammation. In addition, the molecular mechanism of naringin in myocardial fibrosis is mediated by the AT1R/PKC/NOX2/Raf‐1/ERK1/2 signaling pathway.

## Author Contributions

**Juthamas Khamseekaew:** formal analysis (equal), investigation (lead), writing – original draft (lead). **Metee Iampanichakul:** formal analysis (equal), investigation (equal). **Prapassorn Potue:** formal analysis (equal), investigation (equal). **Putcharawipa Maneesai:** formal analysis (equal), investigation (equal). **Panot Tangsucharit:** formal analysis (equal), investigation (equal). **Siwayu Rattanakanokchai:** formal analysis (equal), investigation (equal). **Poungrat Pakdeechote:** conceptualization (lead), supervision (lead), writing – review and editing (lead).

## Conflicts of Interest

The authors declare no conflicts of interest.

## Data Availability

Data presented in this study are available from the corresponding author upon request.
